# Pathogenesis and Management of Intestinal Failure-Associated Liver Disease

**DOI:** 10.1055/a-2545-7370

**Published:** 2025-03-18

**Authors:** Sasha-Jane Abi-Aad, Mark Lovell, Racha T. Khalaf, Ronald J. Sokol

**Affiliations:** 1 Division of Pediatric Gastroenterology, Hepatology and Nutrition, Department of Pediatrics, University of South Florida Morsani College of Medicine, Tampa, Florida; 2 Department of Pathology, Children's Hospital Colorado, University of Colorado School of Medicine, Aurora, Colorado; 3 Department of Pediatrics, Digestive Health Institute, Children's Hospital Colorado, Section of Pediatric Gastroenterology, Hepatology and Nutrition, University of Colorado School of Medicine, Aurora, Colorado

**Keywords:** intestinal failure-associated liver disease, cholestasis, intestinal failure, lipid emulsions, parenteral nutrition-associated liver disease

## Abstract

Long-term parenteral nutrition (PN) has considerably improved the management of intestinal failure (IF) in children and adults, particularly those with short bowel syndrome; however, it carries a significant risk of hepatotoxicity, specifically, intestinal failure-associated liver disease (IFALD), also known as PN-associated liver disease. This review provides an update on the latest understanding of IFALD pathogenesis, emerging therapies, and ongoing challenges in the management of this complication. A number of factors are associated with the development of IFALD. PN lipid emulsions, phytosterol exposure, bacterial dysbiosis, an altered gut–liver axis, and episodes of sepsis disrupt bile acid homeostasis and promote liver inflammation in the active phase of IFALD, favoring the development of PN-associated cholestasis (PNAC) and the more chronic form of steatohepatitis with fibrosis. Based on the identification of pathophysiological pathways, potential therapies are being studied in preclinical and clinical trials, including lipid emulsion modifications; targeted therapies such as Farnesoid X receptor (FXR) and liver receptor homolog 1 (LRH-1) agonists, tumor necrosis factor inhibitors, glucagon-like peptide-2 analogs; microbiome modulation; and supplementation with choline and antioxidants. In conclusion, the pathogenesis of IFALD is complex, and PN dependence and liver injury remain challenging, particularly in patients with IF who cannot advance to enteral nutrition and be weaned off PN.

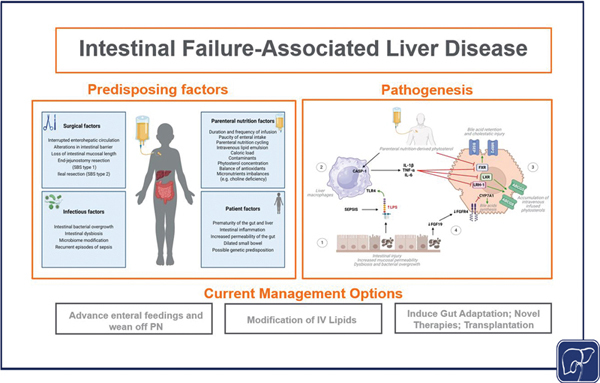

## Epidemiological Aspects and Natural Progression of Intestinal Failure-Associated Liver Disease

**Fig. 1:**
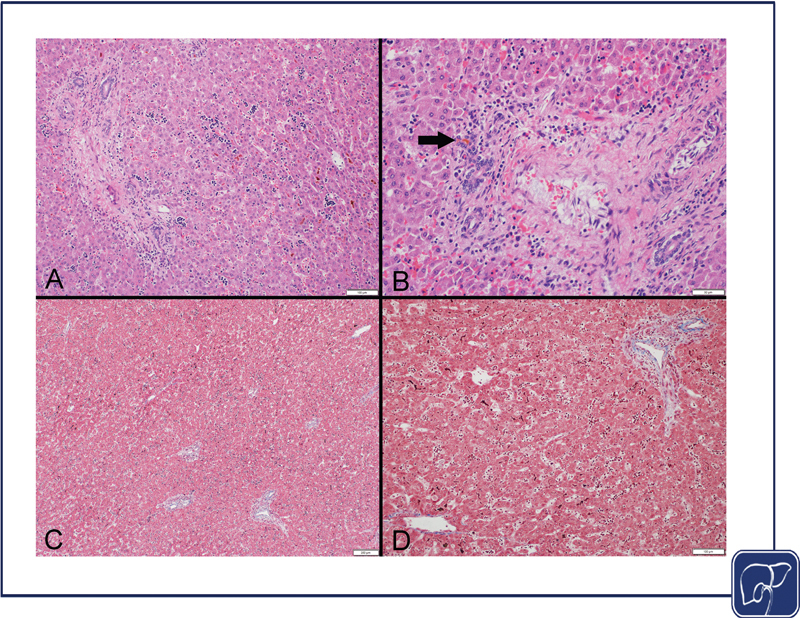
Liver histology of the acute/active phase of IFALD (cholestasis and inflammation). ( **A** ) The hepatic trabecular architecture shows focal pseudoacinar transformation around intracanalicular bile plugs, primarily in a lobular and pericentral distribution with associated extramedullary hematopoiesis. (hematoxylin–eosin, original magnification 40×). ( **B** ) A portal area shows a bile duct plug (arrow). (hematoxylin–eosin, original magnification 100×). ( **C** ) No bridging fibrosis is seen. (trichrome, original magnification 40×). ( **D** ) No periportal, central vein, or sinusoidal fibrosis is seen. (trichrome, original magnification 100×). IFALD, intestinal failure-associated liver disease.

**Fig. 2:**
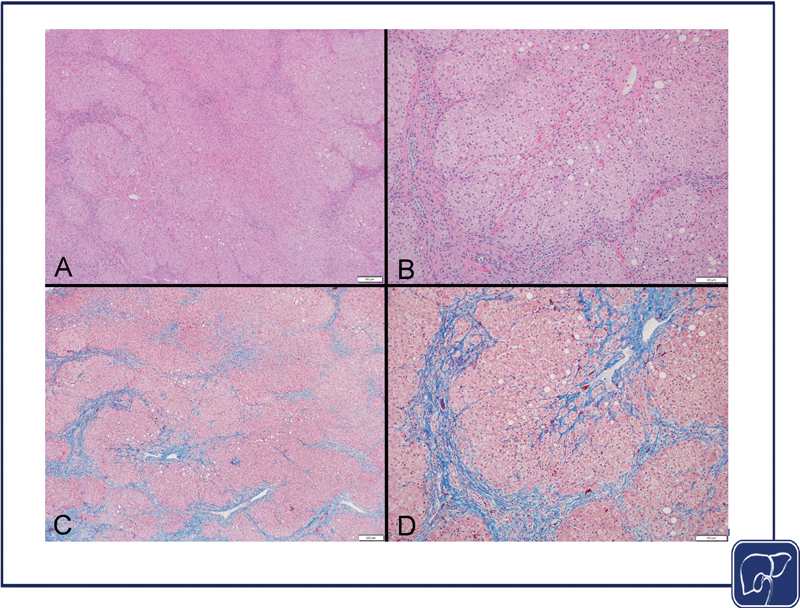
Liver histology of the chronic phase of IFALD (steatosis and fibrosis). ( **A** ) The hepatic architecture is distorted with scattered macrovesicular steatosis, primarily in a lobular and pericentral distribution (hematoxylin–eosin, original magnification 100×). ( **B** ) Hepatocyte swelling is evident with scattered macrovesicular steatosis. Bile ductular reaction is present at the periphery of the portal areas. No cholestasis is evident within the hepatocytes, canaliculi, or bile ducts. (hematoxylin–eosin, original magnification 200×). ( **C** ) Fibrosis is present diffusely with delicate bridging but without cirrhosis. (trichrome, original magnification 40×). ( **D** ) Marked periportal and pericentral fibrosis is highlighted with associated sinusoidal fibrosis and portal-central bridging. (trichrome, original magnification 200×). IFALD, intestinal failure-associated liver disease.

IFALD remains a diagnostic challenge as there are no consensus criteria for its diagnosis, and incidence may vary depending on the varying definitions used in different studies. 3 The overall incidence of IFALD in patients receiving long-term PN is approximately 15 to 40% for adults and 40 to 60% for children, with infants, especially premature newborns with low birth weight, being at a greater risk. 4 The incidence of PNAC is related to the duration of PN; PNAC occurs in >60% of infants receiving PN for >2 months but <16% for PN duration <1 month. 5 In most studies, Phase 1 or active IFALD (characterized by cholestasis and inflammation) is defined as serum direct/conjugated bilirubin greater than 1.0 to 2.0 mg/dL and greater than 20% of total serum bilirubin (TB) in infants and children who have received PN for at least 14 days and in whom other causes of cholestasis have been excluded. 6 Phase 2 or chronic IFALD (characterized by hepatic steatosis and fibrosis) has no generally accepted diagnostic criteria; and because liver function tests do not always correlate with liver histology, liver biopsy, hepatic imaging, or elastography may be the only reliable tools to establish the diagnosis and assess the degree of liver injury. 3 A wide variety of changes may be visible on liver histology including cellular and canalicular cholestasis, portal bile duct plugs, variable portal inflammation, ductular reaction and prominent liver macrophages in Phase I/active IFALD ( Fig. 1 ), and varying amounts of steatosis, hepatocyte swelling, and periportal fibrosis in Phase 2/chronic IFALD ( Fig. 2 ).

Currently, efforts are underway to discover noninvasive methods that accurately predict active IFALD. In a study of 77 children with IF who underwent liver biopsies, those with active IFALD had significantly higher levels of aspartate aminotransferase (AST), alanine aminotransferase (ALT), and γ-glutamyl transferase (GGT) and significantly lower levels of serum citrulline compared to those with chronic IFALD and those without IFALD. A GGT cut-off of 28 U/L, citrulline level of 18.5 μmol/L, and liver stiffness of 5.7 kPa (measured by vibration-controlled transient elastography) had sensitivities for detecting active IFALD of 86%, 83%, and 83%, and specificities of 85%, 71%, and 79%, respectively, with combinations providing higher accuracy. 7 Another biomarker being investigated as a surrogate for liver steatosis is fibroblast growth factor (FGF) 21 (FGF21), which is produced by the liver and regulates glucose and lipid metabolism. In a study of 35 pediatric patients with IF and PN dependence, serum FGF21 levels were significantly higher in those with liver steatosis compared to those without, and these levels correlated with the grade of steatosis. 8 These biomarker findings are promising; however, further studies are needed to assess the external validity of these tests.

In preterm infants without intestinal dysfunction, PNAC can often be reversed by advancing enteral feeding, with PN reduction or PN discontinuation. 5 In contrast, children with IF who remain chronically dependent on PN are more susceptible to IFALD and death. 9 The cholestatic/inflammatory IFALD phase is associated with the ongoing administration of PN, and the steatosis/fibrotic chronic phase may persist even after many years of PN discontinuation. In a study of 38 pediatric IF patients who underwent liver biopsies, Mutanen et al. 10 reported abnormal liver histology in 94% of patients on PN for an average of 74 months and in 77% of patients weaned off PN for an average of 8.8 years. There was significantly increased expression of liver α-smooth muscle actin, liver collagen I, and extracellular matrix adhesion molecules in both patients receiving PN and off PN compared to controls, indicating a persistent proinflammatory and fibrotic state even after weaning off PN; possibly because of ongoing intestinal homeostasis disruption observed in IF. 11 IFALD is a dynamic process evolving over months to years, and PN influences its resolution or progression. Mutanen et al. 7 showed in a study of 77 IF children who underwent liver biopsy at a mean age of 1.7 years, that 48% had acute IFALD, 21% had chronic IFALD, and 31% had no IFALD. After a median follow-up of 2.9 years, 48 patients had a repeat liver biopsy. Among those with initial acute IFALD (25 patients), 36% still had acute IFALD (with most remaining on PN), 29% progressed to chronic IFALD, and 29% regressed to no IFALD (most weaned off PN). Of those with initial chronic IFALD (8 patients), 12.5% had acute IFALD, 37.5% remained chronic (most still on PN), and 62.5% had no hepatotoxicity. Among those with no initial IFALD (14 patients), 21% developed acute or chronic IFALD while remaining on PN, and the rest did not. 7 In a multivariate analysis, Gattini et al. 12 demonstrated that IFALD reduces survival in pediatric IF in the contemporary era (hazard ratio (HR) =3.78, 95% CI 2.18–6.55). Consequently, early recognition and management of IFALD, along with addressing modifiable factors contributing to IFALD development, are essential.

## Pathogenesis of Intestinal Failure-Associated Liver Disease

**Fig. 3:**
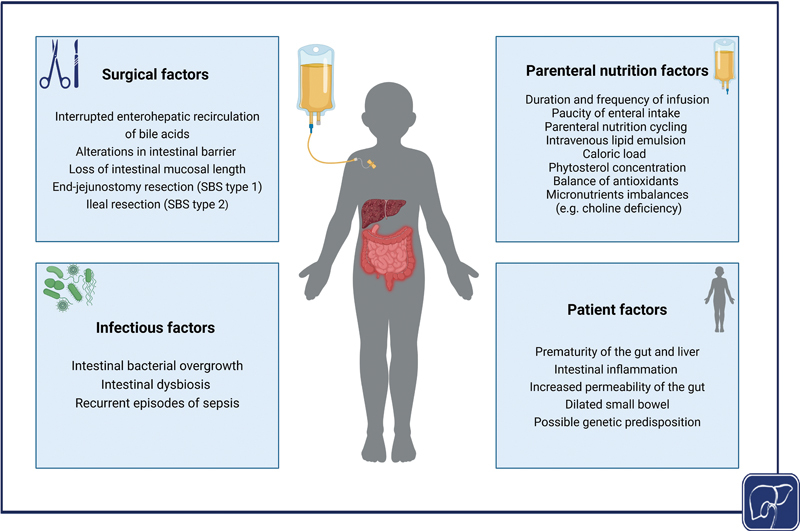
Factors contributing to the pathogenesis of IFALD. Factors contributing to the pathogenesis of IFALD include patient-related, infectious, surgical, and parenteral nutrient factors. Patient-related factors, such as gut and liver prematurity, intestinal inflammation, increased gut permeability, dilated small bowel, and genetic predisposition increase susceptibility to liver injury. Infectious factors, including small intestinal bacterial overgrowth, recurrent sepsis, and intestinal dysbiosis, disrupt the intestinal microbiome, promoting macrophage activation, hepatic inflammation, and cholestasis. Surgical interventions, such as end-jejunostomy and ileal resection leading to short bowel syndrome types 1 and 2, respectively, disrupt the enterohepatic recirculation of bile acids and the gut–liver axis, leading to activation of hepatic inflammatory pathways and alterations in bile acid metabolism. Lastly, PN-related factors, including the type and amount of intravenous lipid emulsions (ILEs), infusion duration and frequency, PN cycling, antioxidant balance, and micronutrient deficiencies (e.g., choline, taurine, glutamine) are associated with IFALD through phytosterol accumulation, oxidative stress, hepatic inflammatory pathways activation, and impaired bile flow. 2 14 Created in BioRender. Abi-Aad, S. (2024). IFALD, intestinal failure-associated liver disease; PN, parenteral nutrition; SBS, short bowel syndrome.

**Fig. 4:**
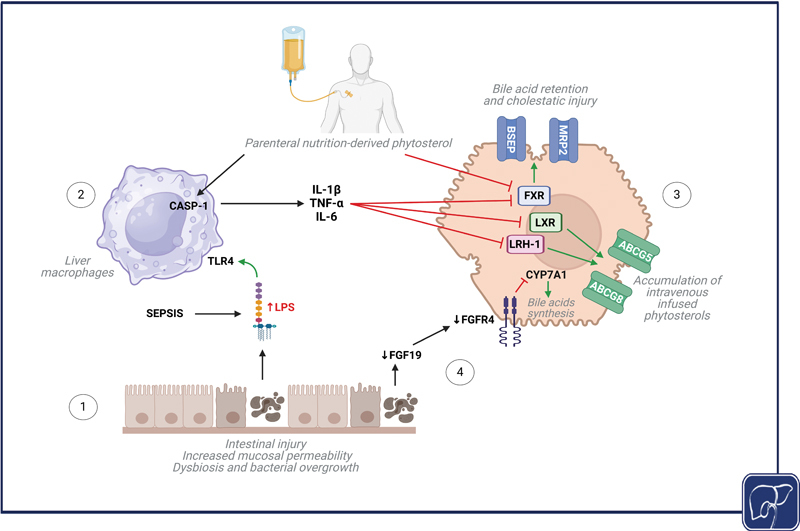
Proposed model of IFALD pathogenesis. (1) Intestinal dysfunction increases mucosal permeability, alters the intestinal barrier, and promotes dysbiosis and small bowel bacterial overgrowth. These changes disrupt the gut–liver axis, facilitating bacterial translocation and absorption of microbial-associated molecular patterns, such as lipopolysaccharides (LPSs), into the portal circulation. Recurrent CLABSI sepsis episodes further enhance LPS release and activation of innate immune pathways. (2) In the liver, LPSs interact with toll-like receptor-4 (TLR4) on hepatic macrophages, activating the inflammasome and an inflammatory cascade and cytokine release (including IL-1β, IL-6, and TNF-α). Binding to their receptors on the hepatocyte, through activation of NFκB signaling, these cytokines downregulate signaling and expression of hepatic nuclear receptors, including Farnesoid X receptor (FXR), liver X receptor (LXR), and liver receptor homolog 1 (LRH-1). (3) The inhibition of FXR, further exacerbated by toxic phytosterol accumulation from parenteral nutrition formulations, downregulates the canalicular bile acid transporter (BSEP) and the conjugated bilirubin transporter, multidrug resistance protein 2 (MRP2), promoting bile acid retention and cholestasis. On the other hand, the inhibition of LXR and LRH-1 downregulates canalicular ATP-binding cassette subfamily G member 5/8 ( *ABCG5* , *ABCG8* ), promoting hepatocyte accumulation of intravenously infused phytosterols. (4) Interruption of the enterohepatobiliary cycle following intestinal injury and resections reduces bile acid reabsorption by enterocytes, leading to diminished FXR activation in enterocytes, decreased fibroblast growth factor 19 (FGF19) production and secretion into the portal circulation, and reduced engagement of fibroblast growth factor receptor 4 (FGFR4) on hepatocytes. This disruption impairs the negative feedback regulating *CYP7A1* , the rate-limiting enzyme in bile acid synthesis, allowing for continued synthesis and accumulation within hepatocytes of toxic bile acids that promote cholestatic liver injury. Created in BioRender. Abi-Aad, S. (2024). CLABSI, central line-associated bloodstream infection; *CYP7A1* , cholesterol 7α-hydroxylase; IFALD, intestinal failure-associated liver disease; PN, parenteral nutrition; SBS, short bowel syndrome.

Patients with PN dependence are more susceptible to hepatic injury due to a combination of patient-related, infection-related, surgical-related, and PN-related factors ( Fig. 3 ). The two phases of IFALD are each mediated by distinct pathophysiological mechanisms since the acute cholestatic/inflammatory phase resolves in the large majority of patients after discontinuation of PN while the chronic steatotic/fibrotic phase often persists long after PN is discontinued. In patients with IF and PN dependence, and in PNAC animal models, cholestasis is associated with the downregulation of canalicular bile acid and phytosterols export transporters, upregulation of the sinusoidal bile acid uptake transporter, and insufficient suppression of bile acid synthesis genes, whereas hepatic steatosis is associated with intestinal microbial dysbiosis, reactive oxygen species (ROS) accumulation, apoptosis, and increased FGF21 ( Fig. 4 ). Although genetic factors may predispose infants to short bowel syndrome (SBS), 13 and hence the need for PN, we are not aware of clear associations of gene variants as risk factors for the development of IFALD, and this hypothesis has not been explored adequately.

## Intravenous Lipid Emulsions and Intestinal Failure-Associated Liver Disease

PN therapies consist of a combination of fatty acids (FAs) from intravenous lipid emulsions (ILEs), amino acids, glucose, vitamins, electrolytes, and micronutrients, which may contribute to IFALD pathogenesis. 14 Factors like high carbohydrate and protein dose, excessive vitamin A, methionine, and manganese, photo-oxidation of vitamins, and deficiencies of taurine, choline, and glutamine, have been associated with the development of IFALD, however, none has been proven causative. 2

ILEs are crucial for PN due to their high caloric density and delivery of essential FAs. The amount and source of ILEs are key in the pathogenesis of IFALD. Soybean oil-based lipid emulsion (SO-LE; such as Intralipid, Fresenius Kabi AG, Bad Homburg, Germany or Nutrilipid, B. Braun, Bethlehem, PA) *,* a commonly used ILE, contains high levels of ω-6-polyunsaturated fatty acids (PUFAs), primarily linoleic acid (LA), a precursor of arachidonic acid (AA) which can be metabolized into proinflammatory eicosanoids, and a substrate for oxidative modification and lipid peroxidation. 15 In contrast, fish oil lipid emulsion (FO-LE), rich in ω-3 PUFAs, contains high amounts of docosahexaenoic acid (DHA) and eicosapentaenoic acid (EPA) with anti-inflammatory properties that can suppress cytokine release from macrophages, hepatocytes, and cholangiocytes. 16 SO-LE provides a significant source of energy and essential FA (LA and α-linolenic acid [ALA]) important for growth and neurodevelopment; however, its high concentration of phytosterols (see below), along with high ω-6:ω-3 ratio and low abundance of antioxidants, makes it more likely to induce IFALD. 17 In contrast, FO-LE with low phytosterols and high antioxidant α-tocopherol levels is less likely to cause liver injury and more likely to reverse cholestasis. In a study of patients with SBS and cholestasis, 44 patients who switched from 3 g/kg/day of SO-LE to the maximum recommended dose of 1 g/kg/day of FO-LE (Omegaven, Fresenius Kabi, Bad Homburg, Germany *)* , experienced a sixfold faster reversal of cholestasis and reduced mortality compared to 49 historical controls who remained on SO-LE (3 g/kg/day). 18 Coconut oil is a source of medium-chain triglycerides (MCTs), which are rapidly metabolized by the liver. Due to their shorter chain and greater hydrophilicity, MCTs are absorbed enterally directly into the hepatic portal vein and undergo β-oxidation. In contrast, long-chain triglycerides (LCTs), like those found in SO-LE, when given enterally, enter the lymphatic system as chylomicrons, reach the systemic circulation, and are deposited in adipose and peripheral tissues. 19 Although some studies have demonstrated a reduction of FA accumulation with MCT use, a study of neonatal piglets who received PN with MCT/LCT-LE for 14 days showed significant increases in TB, direct bilirubin (DB), and AST, as well as inflammatory markers in the blood and ileal mucosa (IL-1B, TNF-α, NF-kβ) compared to neonatal piglets who received FO-LE. On the other hand, piglets who received FO-LE showed higher expression of antioxidant genes such as glutamate-cysteine ligase catalytic subunit, superoxide dismutase 1 (SOD1), and catalase compared to MCT/LCT emulsions. 20

A newer combination of ILE (c-LE) composed of SO (30%), olive oil (25%), MCT (30%), and FO (15%) with added α-tocopherol (SMOF-Lipid™, Fresenius Kabi, Bad Homburg, Germany) is now approved for infants in many countries. Due to its favorable ω-6:ω-3 ratio and reduced phytosterol content, c-LE appears effective in reversing IFALD in most infants, 21 although its effect may be slower than that of FO-LE. In children under 18 years old, the administration of 1.6 g/kg of SMOF-Lipid resulted in a mean decrease of 67.7% in TB levels ( *p* < 0.05) after 4 to 5 months and a significant increase in both height and weight compared to baseline. 22 Term and pre-term neonates with IF who received c-LE experienced a significant decrease in DB levels compared to those who received SO-LE; however, no significant differences were observed in growth parameters. 23 Additionally, a study of low-birth-weight infants (<1,500 g at birth) reported lower mean TB levels after 4 weeks of c-LE compared to SO-LE, reduced incidence of retinopathy of prematurity and intraventricular hemorrhage (complications that may be associated with essential FA deficiency) but no significant difference in z-score for weight, head circumference or crown–heel length. 24 c-LE, which can be given up to 3 g/kg/day in neonates and children <12 years and up to 2.5 g/kg/day for adolescents and adults, may be almost as effective as FO-LE in preventing IFALD with the benefit of providing the full lipid intake similar to SO-LE required for brain growth and development in infants. 25 26

## Plant Sterols (Phytosterols)

Phytosterols play a key role in the development of IFALD. 27 Phytosterols are found in highest amounts in plant-based ILEs (e.g., SO-LE) and very low levels in non-plant-based ILEs (e.g., FO-LE), with c-LE in between. 2 The liver has limited ability to metabolize phytosterols as dietary phytosterols are usually minimally absorbed. In contrast, IV administration of phytosterols provides large loads requiring excretion via biliary sterol transporters ATP-binding cassette subfamily G member 5/8 ( *ABCG5/G8* ; sterolin) located on the canalicular membrane, or export into the intestinal lumen by intestinal epithelia. 28 Impaired biliary excretion, such as in cholestasis and downregulation of *ABCG5/G8* , results in markedly elevated hepatic and circulating concentrations of phytosterols. In several pediatric studies, exposure to phytosterols from PN formulations was indeed associated with cholestatic IFALD and elevated plasma and liver phytosterols' concentrations, including campesterol, stigmasterol, sitosterol, isofucosterol, sitostanol, and cholestane. 28 29 A number of reports indicate that the phytosterols may themselves be causative of PNAC. In a mouse model preclinical study, the administration of a lab-generated low phytosterol SO-LE prevented biochemical and histologic liver injury compared to injury associated with standard SO-LE. 30 In another mouse model of PNAC and intestinal injury, substituting SO-LE with FO-LE prevented liver injury, but the addition of stigmasterol (a known cholestatic phytosterol) to FO-LE-induced liver injury, demonstrating the toxicity of phytosterols in the pathogenesis of IFALD. In addition, hepatic macrophage activation in PNAC is associated with the downregulation of canalicular *ABCG5/G8* sterol transporters, further impairing biliary excretion of phytosterols. The mechanism by which elevated hepatic phytosterol levels cause cholestasis likely involves the interruption of Farnesoid X receptor (FXR) signaling and subsequent downregulation of the canalicular bile acid transporter, bile salt export pump (BSEP; *ABCB11* ; 31 see below). Supporting this proposed mechanism, Mutanen et al. 32 reported that in children with IFALD, ratios of serum stigmasterol to cholesterol and avenasterol to cholesterol correlated with elevated serum bile acids and TB concentrations. These studies and others highlight the association of elevated concentrations of phytosterols with the development of cholestasis in infants and children with IFALD.

## Farnesoid X Receptor

FXR, a nuclear hormone receptor encoded by *NR1H4* , regulates bile acid homeostasis by inducing a small heterodimer partner (SHP) or directly by interacting with promoter regions of genes involved in bile acid synthesis or transport. 33 Of importance related to PNAC and IFALD, infused phytosterols are taken up by hepatocytes and can directly interfere with hepatic FXR signaling, resulting in the downregulation of the canalicular bile acid transporter (BSEP) and the conjugated bilirubin transporter (multidrug resistance protein 2 encoded by the *ABCC2* gene). 2 Phytosterols also activate hepatic macrophages, triggering proinflammatory cytokine release. These cytokines (including IL1-β) bind to receptors on hepatocytes, activating NF-kB signaling, which downregulates the expression of canalicular transporters of bile acids and phytosterols, impairing bile flow and resulting in elevated hepatic bile acid and phytosterol concentrations and cholestasis. 31 In a mouse model with intestinal injury, increased lipopolysaccharide (LPS) absorption combined with IV infusion of SO-LE led to hepatic macrophage activation, IL-1β and TNF-α production, and downregulation of hepatocyte mRNA expression of *Nr1h4* , *Abcb11* , and *Abcc2* , resulting in biochemical and histologic evidence of PNAC. The critical role of hepatic macrophages was demonstrated by the pharmacological blockage of the IL-1β receptor (by anakinra) or genetic deficiency of C-C motif chemokine receptor (CCR2), both of which prevented cholestasis. 34 The critical role of phytosterols was demonstrated in PN-fed mice, which did not develop PNAC when receiving FO-LE; however, when FO-LE was spiked with the phytosterol stigmasterol, hepatic macrophage activation, downregulation of canalicular transporters, and biochemical evidence of cholestasis were all observed. 31

To further maintain bile homeostasis, hepatocyte FXR induces *NR0B2* /SHP, which binds to the cholesterol 7α-hydroxylase ( *CYP7A1* ) gene promoter to prevent the transcription of cholesterol 7α-hydroxylase, the rate-limiting enzyme of bile acid synthesis, thus preventing excessive bile acid accumulation in hepatocytes. In IFALD, this mechanism is dysregulated. In a study involving 33 pediatric patients with SBS and PN dependence, 35 hepatic mRNA expression of *CYP7A1* was significantly elevated in both PN-dependent and weaned patients, compared to controls, suggesting persistent bile acid synthesis in the face of cholestasis, further worsening cholestatic injury. Similarly, other hepatocyte bile acid uptake transporters were upregulated in SBS and PNAC such as sodium taurocholate co-transporting polypeptide (NTCP/ *SLC10A1* ) for basolateral uptake of conjugated bile acids and organic anion-transporting polypeptide 1B1 (OATP1B1/ *SLCO1B1* ) for basolateral uptake of unconjugated bile acids, while export transporters like basolateral bile acid exporter multidrug resistance-associated protein 3 were downregulated. The combined effect was further promoting bile acid accumulation in hepatocytes. 35 This dysregulation of bile acid homeostasis may result in SBS patients, at least in part, from insufficient ileal secretion of FGF19, which normally downregulates *CYP7A1* expression in the liver. 35

## Liver X Receptor

Liver X receptor (LXR) is a nuclear receptor encoded by *Nr1h3* that regulates sterol metabolism through its binding to the promoter of *ABCG5/8* . These transporters are essential for canalicular export of infused phytosterols and are not upregulated appropriately in mouse models and children with PNAC. In an age-matched case–control study, infusion of PN in children with IF was associated with decreased expression of LXR, ABCG5, and ABCG8, as well as FXR and bile acid transporters, 36 which was related to hepatic inflammatory infiltrates, similar to the findings in the PNAC mouse model.

## Liver Receptor Homolog 1

Liver receptor homolog 1 (LRH-1), encoded by *Nr5a2* , is another nuclear receptor that regulates sterol metabolism and bile acid synthesis. Ghosh et al. 33 found in a mouse PNAC model that LRH-1 activates the promoter region of *ABCG5* , *ABCG8* , and *Cyp7A1* to maintain bile acid homeostasis. PN led to an NF-kB-dependent disruption of LRH-1 signaling that was associated with the downregulation of these bile transporters and PNAC. In a study in cultured hepatocytes, LRH-1 expression was decreased after exposure to phytosterols (stigmasterol + sitosterol) with or without the addition of LPS and with LPS alone, resulting in the downregulation of *ABCG5/8* and stimulation of *CYP7A1* and, subsequently, bile acid accumulation and PNAC. Furthermore, this process was mediated by IL-1 and NF-kβ signaling. An LRH-1 agonist, 1,2-dilauroyl-sn-glycero-3-phosphocholine (DLPC), prevented PNAC in this mouse model, suggesting this pathway as a possible therapeutic target. 37

## Ileal Fibroblast Growth Factor 19 Signaling and Intestinal Failure-Associated Liver Disease

FGF19, secreted by the ileum and upregulated in the human liver during cholestasis is an important negative feedback regulator of bile acid synthesis in humans. When bile acids secreted by the liver are reabsorbed in the intestine, they activate enterocyte FXR, which promotes the synthesis and release of FGF19 into the portal circulation. When FGF19 reaches the liver, it binds to FGF receptor 4 (FGFR4) on hepatocytes and inhibits *CYP7A1* , thus controlling bile acid synthesis. In patients with intestinal atresia, ileal resections, or significant ileal inflammation, many of whom require long-term PN, there may be insufficient intestinal synthesis and secretion of FGF19 and, thus, less negative feedback on bile acid synthesis. In a study of 52 patients with IF and PN dependence, circulating FGF19 levels were significantly lower compared to controls, particularly in those with total ileal and ileocecal valve resection and those with histological evidence of IFALD or portal inflammation. 38 Several factors may lead to lower FGF19 levels. Patients with IF may be unable to adequately reabsorb bile acids due to disruption in the enterohepatic circulation caused by mucosal inflammation or surgical removal of the distal small intestine, which expresses apical sodium-dependent bile acid transporters responsible for conjugated bile acid absorption. 35 The lower amount of bile acids reabsorbed into enterocytes would lead to insufficient stimulation of enterocyte FXR and inadequate FGF19 synthesis and secretion, impairing the negative feedback on hepatic bile acid synthesis. The overall result is excessive bile acid production during cholestasis and accumulation of potentially toxic bile acids in hepatocytes. 38 In this regard, Xiao et al. 39 recently reported that a serum FGF19 level below <107 pg/mL was a significant predictor of cholestasis and liver stiffness in patients with IF, with an OR of 13.184 (95% CI, 4.661–37.289, *p* < 0.001). Based on the effect of FGF19 on hepatic bile acid synthesis, FGF19 analogs have been explored as potential therapies to reduce hepatobiliary injury in other cholestatic diseases, but not in IFALD, to the best of our knowledge. 40

## Alterations of Intestinal Barrier Function

Intestinal barrier function may be impaired in IF through several mechanisms, including inflammation common in SBS, small intestine bacterial overgrowth (SIBO) and dysbiosis, portal hypertension, and immaturity. The resulting increased absorption of microbial-associated molecular patterns (MAMPs), such as LPS derived from the intestinal microbiota, likely plays an important role in hepatic macrophage recruitment and activation in IFALD. 2 Moreover, the intestine has an autoregeneration capacity mediated by intestinal stem cells (ISCs), which express the leucine-rich repeat-containing G protein-coupled receptor 5 (LGR5). To maintain gut integrity and intestinal repair, a high abundance of LGR5+ ISC is required. The renewal of these cells is dependent on mitochondrial FA oxidation, a process regulated by FXR. Using patient-derived organoid models obtained from intestinal biopsies of pediatric SBS with ( *n* = 8) and without IFALD ( *n* = 10), Zhao et al. 41 reported significant reductions of LGR5+ ISC population and impaired FXR signaling in those with IFALD. The addition of T-βMCA, an FXR antagonist, exacerbated enterocyte damage and downregulated FA oxidation gene expression. In contrast, the restoration of FXR function by an FXR agonist did not result in enterocyte proliferation or ISC recovery. These findings suggest that FXR may play a role in maintaining gut integrity through the regulation of FA oxidation; however, further studies are needed to understand how this may be affected in IFALD.

## Microbiome Signature Changes

In patients with SBS, SIBO, small bowel dilation (>30 mm diameter), absence of the ileocecal valve, and altered gut permeability can lead to intestinal dysbiosis, increasing MAMPs and bacterial translocation. 2 Patients with IF experience significant gut microbiota changes, including reduced microbial diversity and overabundance of LPS-enriched bacteria like Bacilli (especially Lactobacilli) and Proteobacteria (especially Enterobacteriaceae), likely contributing to IFALD disease progression. 42 43 When the gut barrier is disrupted, the liver is the first organ to receive intestinally derived MAMPs, toxins, and microorganisms. 44 For example, Proteobacteria expressing LPS can enter portal circulation if the intestinal mucosal barrier is compromised. 43 LPS can then bind to toll-like receptor 4 (TLR4) in hepatic macrophages, which signals downstream activation of proinflammatory cytokine expression and secretion and subsequent hepatocyte injury and cholestasis. In a mouse model with intestinal injury and total parenteral nutrition (TPN), genetically impaired TLR4 signaling abrogated the LPS-induced IL-6 expression in hepatic macrophages. 45 Moreover, mice treated with an antibiotic mixture that reduced intestinal bacteria by >97% did not show increased expression of hepatic proinflammatory cytokines (IL-6 and TNF-α) and experienced a significant reduction in liver injury. 45 In a preterm piglet model with TPN-induced IFALD, liver RNA sequencing revealed upregulated inflammatory pathways (NF-kβ, TNF-α, and TLRs), and cytokines compared to controls, while treatments with corticosteroids, anti-TNF antibody, NF-kβ inhibitor or cyclooxygenase-2 inhibitor downregulated these pathways, supporting that early IFALD is predominantly an inflammatory liver disease. 46 In addition, LPS-induction of TNF-α expression in mouse hepatocytes led to a decrease in mRNA expression of genes regulating bile acid and bilirubin export ( *Nr1h4* /FXR, *Abcb11* , *Abcc2),* as well as reduced expression of sterol transporter pathway genes ( *Nr1h3* /LXR, *Abcg5,* and *Abcg8* ). Finally, knockout of TNF receptor in the PNAC mouse model or treatment with anti-TNF-antibody (infliximab) restored canalicular bile acid and sterol transporter expression and resulted in lower serum levels of bile acids, bilirubin, ALT, and AST. 47 Taken together, these studies indicate a key role for the gut microbiome and its products (and the gut–liver axis) in the pathogenesis of PNAC and IFALD.

## Hepatic Steatosis and Novel Transcriptional Factors

Possibly one of the causes of hepatic steatosis in IFALD relates to the transcription factor HES6, which regulates FA metabolism and oxidation. *Hes6* expression was significantly decreased in a mouse model of 7 days of TPN compared to control mice. *Hes6* expression was found to be positively correlated with several taurine-conjugated bile acids but negatively correlated with hepatic triglycerides level (R = −0.68). Thus, HES6 may play a significant role in the development of hepatic steatosis during PN. 48

## Oxidative Stress and Apoptosis

*Fas* -mediated apoptosis has also been proposed as an important pathway leading to PNAC. In a mouse model of intestinal injury and 14 days of PN, *Fas* expression was induced in hepatocytes following IL-1β treatment and in hepatic mononuclear cells following LPS injection but was significantly decreased in IL-1β knockout mice, demonstrating the role of IL-1β signaling in *Fas* -mediated apoptosis. This rise in *Fas* expression was associated with elevated expression of caspase 8 and cleaved caspase 3 levels indicative of apoptosis. On the other hand, the IL-6-STAT pathway, which normally protects against liver damage, was also upregulated in the PNAC mouse model following IL-1β exposure, indicating a remaining protective effect in PNAC. The addition of GW4064 (an FXR agonist) further activated IL-6-STAT by binding to its promoter region, reducing expression of *Fas* , caspase 8, and cleaved caspase 3, thus providing a potential therapeutic target to overcome PNAC. 49 In addition, the downregulation of several NADH dehydrogenase subunits (mitochondrial complex I), particularly NADH:ubiquinone oxidoreductase core subunit S1 (NDUFS1), has been associated with IFALD in both human and rat models. Disruption of mitochondrial oxidative phosphorylation in hepatocytes resulted in elevated ROS production and liver injury. Moreover, antioxidant treatment with MitoQ reduced ROS levels and significantly improved liver damage, cholestasis, and inflammation. Similarly, the restoration of NDUFS1 expression improved liver injury, 50 indicating potential pathways to explore that might alleviate liver injury.

## Treatment and Prevention Strategies

The overall goal of managing patients with IF is to wean them from PN and establish complete enteral nutrition. However, there is a subset of children and infants with IF who cannot be tapered from PN and require care best delivered by a multidisciplinary team 1 that may employ strategies involving pharmacologic, nutritional, and surgical interventions to stabilize, reverse, and prevent liver injury.

### Progression to Enteral Autonomy

Early advancement of enteral feedings, with the ultimate goal of full enteral feeding, if possible, will improve outcomes of patients with IF and IFALD. 51 Bolus enteral feedings may have an advantage over continuous tube feedings by stimulating intestinal motility, promoting mucosal hyperplasia and hypertrophy, reducing SIBO/dysbiosis, and increasing gallbladder contractility and bile flow, all of which may reduce the risk of cholestasis. 52 53 However, a significant proportion of infants and children with IF cannot be weaned off PN 54 and only tolerate a limited amount of enteral nutrition and thus require additional approaches.

### Lipid Emulsion Dose Reduction

Multiple studies show that reducing the total daily doses of ILEs may delay IFALD onset and reduce its severity. 55 56 In neonates (≥36 weeks gestational age) with GI surgical disorders, administration of 1 g/kg/day of SO-LE for 6 months resulted in the same IFALD rate as 2 g/kg/day, but the weekly increase in DB was slower with the lower dose. 17 Another study in neonates ≥5 days old with GI disorders found no difference in cholestasis between low (1 g/kg/day) and traditional doses (3 g/kg/day) of SO-LE (30% vs. 38%, *p* = 0.7), but similarly showed a slower increase in DB with lower doses. 57 In contrast, others have shown that reducing the total amount of SO-LE to 1 g/kg/day can reverse cholestasis. 58 Postsurgical neonates receiving 1 g/kg/day of SO-LE had a 21% less incidence of IFALD compared to those receiving traditional SO-LE dose of 2 to 3 g/kg/day. 59 Cyclic PN and interruptions in delivery for several hours daily are recommended to decrease the risk for cholestasis, although this approach brings with it a risk of hypoglycemia, and there are limited data in children supporting a significant improvement in IFALD. 60 61 However, in adult patients with IFALD, cyclic PN for an average of 12 days significantly improved AST, GGT, and TB, but no differences were observed in other liver tests. 60 Because neonates require appropriate nutritional and lipid intake to promote brain growth, 27 providing a suboptimal ILE dosage has the potential to lead to impaired brain growth or neurodevelopmental outcomes. In fact, a study in newborn piglets reported that a half dose of either SO-LE or FO-LE in TPN resulted in lower brain weight after 2 weeks compared to a full dose of either lipid emulsion. 62 Another study on neonatal piglets reported reduced brain weight with the use of low-dose FO-LE compared to a high dose of SO-LE. 62 However, to date, there have not been any studies showing impaired neurodevelopmental outcomes in human infants related to various doses and types of ILEs. Finally, there is concern that low-dose ILE therapy could increase the risk of essential FA deficiency, which could result in poor growth and increased risk of infection, thrombocytopenia, hepatitis, and hypertriglyceridemia. 63 However, this has not been observed in infants who have received 1 g/kg/day of ILE. 63

### Alternative Lipid Sources

The safety and efficacy of FO-LE in patients with IFALD led to the Food and Drug Administration (FDA) approval of Omegaven™ in 2018. Gura et al. 64 demonstrated that children with PN dependence and cholestasis who switched to 1 g/kg FO-LE had an adjusted HR of 6.8 (95% CI: 1.7–27.8) for reversing cholestasis, with lower rates of liver transplant and death compared to a historical matched cohort who did not receive FO-LE, and no adverse events, such as coagulopathy or essential FA deficiency, were reported. Another study demonstrated a 75% reversal of PNAC in cholestatic infants after 3 to 6 months of 1 g/kg/day FO-ILE compared to only 6% in those continuing SO-ILE at 2 to 3 g/kg/day. 65 Recent guidelines for pediatric and preterm PN have been published to guide clinicians with ILE use. 66 67 FO-LE is not entirely without potential risk; high concentrations of ω-3 PUFAs (DHA and EPA) can increase bleeding risk due to competition with AA for cyclooxygenase, potentially reducing thromboxane A2 and impairing platelet aggregation. However, a comparative study found that the use of SO-LE in children with IFALD was associated with a higher bleeding risk than FO-LE, possibly due to the greater severity of liver dysfunction over time in those receiving SO-LE. 15 Because data are still lacking and FO-LE contains a low concentration of essential FAs and AA, it is currently not recommended for patients without IFALD. 63 Although FO-LE reverses cholestasis and liver inflammation (Phase 1 IFALD), hepatic fibrosis (Phase 2) may persist for years even after switching to FO-ILE, 68 suggesting that persistent hepatic fibrosis may be driven by intestinal dysfunction rather than PN administration in IFALD patients.

c-ILE (combined intravenous lipid emulsion; SMOFLipid™), another alternative, was FDA-approved for adults in 2016 and pediatric patients in 2022, including term and preterm neonates. Newer formulations of ILEs are emerging and showing promise in preclinical models; however, studies in adults and children are still needed to evaluate the efficacy and safety of these products. Vegaven™ (VV), a novel ILE, contains 30% of 18-carbon ω-3 FA, particularly ALA and stearidonic acid. In a mouse preclinical model, the administration of VV-TPN increased IL-10 and decreased IL-6 levels compared to standard LEs (SO-IL and Omegaven/FO-IL). VV also amplified the expression of LPS-binding protein, which detoxifies endotoxin in the liver, and upregulated the hepatic nuclear peroxisome proliferator-activated receptor α (PPARα), a transcription factor associated with FA oxidation and protection against steatohepatitis. The VV formula was shown to have greater resistance to oxidative modification compared to standard lipid emulsions. 69 Although these preclinical studies appear promising, it must be emphasized that VV has not been tested in infants and children and is not FDA-approved at this time, and thus requires further testing in humans to assess effectiveness and safety.

## Pharmacotherapies

Choline is an essential nutrient involved in the formation of phosphatidylcholine, the major phospholipid transported into bile by canalicular MDR3. Previous studies have reported a decrease in plasma-free choline levels in older children and adult patients receiving long-term PN. 70 In a rat model, the addition of choline to PN improved liver function tests, decreased hepatic triglycerides levels, reduced ROS by 37%, and upregulated genes involved in FA oxidation, including hepatic PPARα and carnitine palmitoyl transferase I (CPT1). 71 These findings suggest that choline supplementation may protect against liver inflammation and steatosis, and clinical trials in humans are in progress. Another proposed intervention to help decrease oxidative stress and protect from liver injury is the administration of exogenous antioxidants in PN. Indeed, the generation of ROS and apoptosis are stimulated by the exposure of isolated rat hepatocytes to hydrophobic bile acids, and this can be reversed by a variety of antioxidants. 72 Moreover, mitochondrial dysfunction appears to be responsible for ROS generation and induction of cell death pathways in hepatocytes. 72 Supplementation with vitamin E prevented both mitochondrial dysfunction and ROS generation in rats given IV hydrophobic bile acids. 73 FO-LE and c-LE are supplemented with higher amounts of α-tocopherol than SO-LE to prevent peroxidation of the PUFAs within the emulsions, although it is not clear if the α-tocopherol is responsible for less cholestasis when these emulsions are administered compared to SO-LE. Supplementation of glutathione in guinea pigs with PN-dependence prevented hypermethylation and inactivation of detoxification genes and protected the liver against oxidative stress induced by lipid peroxidation. 74

The administration of dexamethasone in preterm neonates before initiating PN may prevent the occurrence of PNAC. Among 78 preterm neonates receiving PN, the prior use of dexamethasone treatment for non-PNAC indications was significantly less common in those who developed PNAC compared to those who did not. Following a logistic regression, dexamethasone use was identified as a protective factor with an OR = −2.739 ( *p* = 0.017). 75 Further studies are needed to examine the potential benefit of dexamethasone in preventing or treating PNAC in infants.

## Medical Induction of Intestinal Adaptation

Teduglutide, a glucagon-like peptide-2 (GLP-2) analog, can induce intestinal adaptation in patients with SBS and reduce the need for PN and intravenous volume support. 76 The efficacy of this therapy led to its FDA approval for children and adults with SBS and PN dependence. Additionally, a recent study in patients with SBS and PN dependence without preexisting liver diseases demonstrated significant mean changes from baseline values to 24 weeks for AST, ALT, and TB among those receiving teduglutide ( *n* = 109) compared to controls ( *n* = 59). However, no changes were observed in RIALP, GGT, platelets, Fibrosis-4 (FIB-4) score, and AST to Platelet Ratio Index (APRI). 77 An inhibitor of dipeptidyl peptidase-4 (DPP-4) (DPP4-I), an enzyme involved in glucose regulation and incretin degradation, is another therapy being investigated for use in patients with SBS; however, the results are still inconclusive. In a rat model of SBS with normal enteric feeding, DPP4-I administration significantly increased villous height in the jejunum and ileum, improved liver blood tests (lower AST, ALT levels), and reduced histological evidence of liver injury reflected in lower hepatic SAF (steatosis, activity, fibrosis) scores, compared to saline-infusion controls. However, no differences were noted for other liver markers. 78

## Emerging New Therapies

Newer targeted therapies have been proposed to potentially delay, reverse, or prevent PNAC and IFALD. FXR ligands (such as GW4064, obetacholic acid [OCA; FDA-approved for use in adults with primary biliary cholangitis], and others) are effective in reversing or preventing PNAC in mouse and piglet models. In a mouse model of intestinal injury and TPN, treatment with GW4064 resulted in a significant reduction in serum AST, ALT, bilirubin, and total serum bile acids and considerable restoration of FXR activity, along with the upregulation of its targets *Abcb11* , *Abcc2* , *SHP* , and *ABCG 5/8* compared to controls. 79 Similarly, treatment with anti-TNF inhibitors (infliximab) or anti-IL-1β receptor therapy (anakinra) in the same mouse model significantly upregulated the expression of *Abcb11* , *Abcc2* , *Abcg5/8* , *Nr1h3* , and *Nr1h4* and prevented cholestasis. 47 Recently, in a newborn piglet model receiving TPN, administration of OCA was shown to prevent PNAC. 80 These findings suggest that FXR analogs or TNF-α and IL-1β signaling inhibitors could serve as potential therapeutic options to reverse PNAC.

## Microbiome Therapies

It is well-established now that SBS predisposes patients to small bowel dilation, favoring dysmotility, inflammation, intestinal dysbiosis, and SIBO, which promote barrier dysfunction and bacterial and MAMP translocation. These processes are likely involved in the pathogenesis of PNAC and IFALD. 81 Strategies to prevent or reverse IFALD are being tested and will leverage our knowledge of the gut–liver axis. Short-chain FAs, including acetate, propionate, and butyrate, provide energy for enterocytes and maintain gut integrity. Butyrate, produced by fermenting dietary carbohydrates by Ruminococcaceae or Lachnospiriaceae (depleted in the intestines of PN-dependent SBS patients), has been shown in preclinical studies to alleviate IFALD by maintaining the intestinal barrier and reducing bacterial translocation to the liver. 44 Other approaches are directed at modifying the gut microbiota through antibiotics, prebiotics, probiotics, or synbiotics. Probiotics, such as *lactobacillus plantarum* ( *L* . *plantarum* ) and *Lactobacillus johnsonii* ( *L. johnsonii* ), were shown to prevent IFALD in preclinical studies. In newborn piglets receiving PN, those who received PN + *L. plantarum* versus PN controls had better intestinal integrity with increased tight junction protein expression ( *TPJ1* , *CDH1* , *CLDN1* ), higher expression of intestinal FGF 19 and SHP, and lower hepatic *CYP7A1* expression associated with reduced hepatic steatosis and lobular inflammation. 82 Similarly, rats treated with PN + *L. johnsonii* had lower levels of the bile acid glycochenodeoxycholic acid, a known inducer of apoptosis, and showed less hepatocyte apoptosis compared to rats fed with TPN only. However, the administration of *L. johnsonii* did not reduce the hepatic expression of *CYP7A1* . There are no reports to date of improved hepatic outcomes using these various modalities to modify the gut microbiome in infants or children with IFALD or PNAC. Importantly, the safety of these pre- and probiotics in humans, particularly children, with IFALD remains unknown, thus, further studies in humans will be required to assess the potential efficacy and safety of pre- and probiotics in IFALD. 83

## Prevention of Central Line-Associated Bloodstream Infections

Recurrent central line-associated bloodstream infections (CLABSIs) are linked to higher risks for the development of IFALD; therefore, implementing preventive strategies is crucial for reducing the incidence and severity of IFALD. 1 84 85 CLABSIs are likely involved in IFALD through the release of LPS and other MAMPs into the systemic circulation from gram-negative infections, which can activate macrophages and other inflammatory cells when they reach the liver, inducing downregulation of hepatocyte bile and sterol transporters, leading to cholestasis. 45 The use of ethanol locks or antimicrobial taurolidine–citrate locks and meticulous care of central venous catheters have been shown to reduce the incidence of CLABSIs and are employed by most multidisciplinary IF care teams. 86 87

## Surgical Lengthening Procedures

Surgical lengthening and tapering procedures and restoration of intestinal continuity with the closure of intestinal stroma, especially at an early age (<1 year), can improve enterohepatic circulation and restore intestinal motility, which may reduce dysbiosis. Importantly, these procedures may increase the surface area of intestinal mucosa, leading to faster advancement of enteral feedings and attainment of enteral autonomy. Although these approaches are important in managing infants and children with SBS, trials have not demonstrated the direct benefits of non-transplant surgery in the management and prevention of IFALD. 2

## Organ Transplantation

The need for small bowel or multivisceral transplantation in managing IF and SBS has decreased in the United States over the past two decades. This is due to advancements in multidisciplinary care, including optimized nutrition, improved ILE, prevention of CLABSIs, use of GLP-2 agonists, and intestinal lengthening procedures. 88 Organ transplantation is usually reserved for severe IFALD cases unresponsive to other interventions and associated with end-stage liver disease. The organ transplantation options include isolated small bowel transplantation for patients with severe IF complications or loss of IV access but with minimal or no signs of IFALD, isolated liver transplantation for portal hypertension and cirrhosis but with adequate intestinal adaptation, and combined liver–intestine transplants (or multivisceral transplants) for patients with complete intestinal aganglionosis, intestinal pseudo-obstruction, or severe portal hypertension. 89 However, the outcome and prognosis of IF patients requiring organ transplantation are not optimal. The Pediatric IF Consortium reported on 272 infants with IF and observed an incidence of enteral autonomy of 47%, death of 27%, and intestinal transplantation of 26%. 90 More recently, Norsa et al. 91 reported that an estimated 5-year survival rate for pediatric patients with neonatal very SBS was 68%, with survival following intestinal transplant of 60% compared to a 100% survival rate for those who remained on long-term PN without life-threatening complications. Despite strong international efforts, outcomes following intestinal transplants fall short of those for other solid organ transplants, mandating doubling down on efforts for continued improvements in medical, nutritional, and surgical management of IF.

## Conclusion

IFALD, including PNAC and steatohepatitis, is a life-threatening complication of long-term TPN. The pathogenesis of IFALD is complex, but ongoing research is clarifying the role of the gut–liver axis and innate immunity, IV lipid emulsions, dysregulation of bile acid metabolism, and intestinal dysbiosis. Innovative medical therapies are emerging, and the need for organ transplants is declining. However, PN dependence and liver injury remain a challenge, especially for patients with IF unable to be weaned off PN and receive enteral nutrition.

## Concluding Remarks

There is a need to establish consensus criteria for diagnosis and categorization of the two phases of IFALD (cholestatic/inflammatory and steatotic/fibrotic), and search for noninvasive diagnostic tools or biomarkers to evaluate IFALD in infants and children with IF. The identification of effective preventative and therapeutic strategies is a high priority. Finally, future research should also focus on novel therapeutics to enhance intestinal adaptation to allow for advancing enteral feedings and reduction of the need for PN in people with SBS.

## Notes

Lay Summary Intravenous (IV) nutrition, also called parenteral nutrition (PN), is a critical therapy for children and adults with complicated intestinal diseases who are unable to tolerate the oral intake of food and fluids. Unfortunately, long-term PN poses a significant risk of liver injury, a condition known as intestinal failure-associated liver disease (IFALD), which can cause inflammation and fat deposits in the liver, leading to scarring and eventually liver failure. The interplay between injured intestines, the bacteria that inhabit our intestines (microbiome), and components of the PN fluids are believed to cause IFALD. This review summarizes recent advances in our understanding of the mechanisms that lead to IFALD and potential therapies currently being studied to treat or prevent this disease, including nutritional approaches, medications, and changing the composition of gut bacteria. In conclusion, the management of patients with IFALD remains a challenge in patients with severe bowel disruption who remain dependent on PN.
